# A Strategy for Uncovering the Serum Metabolome by Direct-Infusion High-Resolution Mass Spectrometry

**DOI:** 10.3390/metabo13030460

**Published:** 2023-03-22

**Authors:** Xiaoshan Sun, Zhen Jia, Yuqing Zhang, Xinjie Zhao, Chunxia Zhao, Xin Lu, Guowang Xu

**Affiliations:** 1 CAS Key Laboratory of Separation Science for Analytical Chemistry, Dalian Institute of Chemical Physics, Chinese Academy of Sciences, Dalian 116023, China; 2 University of Chinese Academy of Sciences, Beijing 100049, China; 3 Liaoning Province Key Laboratory of Metabolomics, Dalian 116023, China; 4 Department of Cell Biology, College of Life Sciences, China Medical University, Shenyang 110122, China; 5 Zhang Dayu School of Chemistry, Dalian University of Technology, Dalian 116024, China

**Keywords:** metabolomics, direct-infusion, high-resolution mass spectrometry, formula assignment, reaction network

## Abstract

Direct infusion nanoelectrospray high-resolution mass spectrometry (DI-nESI-HRMS) is a promising tool for high-throughput metabolomics analysis. However, metabolite assignment is limited by the inadequate mass accuracy and chemical space of the metabolome database. Here, a serum metabolome characterization method was proposed to make full use of the potential of DI-nESI-HRMS. Different from the widely used database search approach, unambiguous formula assignments were achieved by a reaction network combined with mass accuracy and isotopic patterns filter. To provide enough initial known nodes, an initial network was directly constructed by known metabolite formulas. Then experimental formula candidates were screened by the predefined reaction with the network. The effects of sources and scales of networks on assignment performance were investigated. Further, a scoring rule for filtering unambiguous formula candidates was proposed. The developed approach was validated by a pooled serum sample spiked with reference standards. The coverage and accuracy rates for the spiked standards were 98.9% and 93.6%, respectively. A total of 1958 monoisotopic features were assigned with unique formula candidates for the pooled serum, which is twice more than the database search. Finally, a case study of serum metabolomics in diabetes was carried out using the developed method.

## 1. Introduction

Direct infusion combined with high-resolution mass spectrometry (DI-HRMS) is an attractive alternative for high-throughput metabolomics analysis due to its simplicity, high resolution, high mass accuracy, and time-saving [1,2]. In metabolomics analysis, nanoelectrospray ionization (nESI) DI-HRMS provides similar discrimination capabilities to conventional liquid chromatography-mass spectrometry (LC-MS) while greatly reducing the total analysis time [3,4]. DI-HRMS approach has been widely applied in various metabolomics studies [5,6,7], such as urinary metabolic profiling in human epidemiological investigations [8], metabolite analysis for the diagnosis of inborn errors of metabolism [9,10,11], and serum metabolomics analysis in diabetes [12].

Metabolite annotation is widely recognized as a critical part of metabolomics analysis. Accurate molecular formula assignment is a prerequisite for identifying unknown metabolites [13]. Typically, metabolite annotation relies on metabolome database searching using accurate mass, where the extent of database coverage determines the annotation performance [14,15,16]. Sarvin et al. [17] recently analyzed the *m*/*z* value distribution of ions and identified optimal scan ranges, resulting in a ~50% increase in feature detection compared to conventional spectral-stitching FI-MS [18]. However, this study employed publicly available databases, such as the LIPID MAPS Structure Database (LMSD) for lipidomics data [19] and the Human Metabolome Database (HMDB) for metabolomics data [20,21], and only 564 out of 3233 features in metabolomics data and 401 out of 3339 features in lipidomics data obtained putative annotations with a 5 ppm mass tolerance, respectively. Alternatively, the stochastic molecular generator method, which incorporates chemical constraints (e.g., high mass accuracy, limited element number/species, and high-fidelity isotopic pattern), allows reliable formula assignment without database limitations [22,23,24]. But this approach is not suitable for high-resolution mass spectrometry analysis, especially for low-abundance and high-mass MS signals, for which multiple formula candidates may match [25,26,27].

Metabolites serve as substrates and products of vital biological reactions [28,29], so it is very promising to exploit the reaction relationships among them for metabolome annotation. The approach of utilizing mass differences has been applied for molecular formula assignment in ultra-high-resolution mass spectrometry [30,31,32]. In this method, spectral features are represented as nodes, while pre-defined metabolic reactions, as reflected by mass differences, serve as edges connecting these nodes [33,34]. High mass accuracy (<1 ppm) is crucial for minimizing the number of candidate molecular formulas and possible reaction types. Nevertheless, it is difficult to achieve in high-resolution mass spectrometry methods [35]. To address this challenge, Chen et al. [36] proposed a global network optimization approach, NetID, which annotates formulas for as many MS features as possible, including adducts, fragments and isotopes of metabolites in untargeted LC-HRMS data. In NetID seed nodes were limited to the detected features and assigned by matching peaks to the HMDB with a mass tolerance of 10 ppm, it is especially important to use highly reliable metabolite formulas as seed nodes [37].

In this work, a serum metabolome characterization method was proposed to fully explore the potential of DI-nESI-HRMS. To enhance the coverage and accuracy of unique formula assignment, a reaction network was constructed and endogenous metabolites from the HMDB served as initial seed nodes. A scoring system was established to further screen reliable molecular formulas from the possible candidates based on the topological relationship in the reaction network, mass accuracy, and isotopic fine structure. Finally, the developed method was applied to a high-throughput study of serum metabolomics in diabetes.

## 2. Materials and Methods

### 2.1. Chemicals

LC-MS grade formic acid was purchased from J&K Scientific Ltd. (Beijing, China). HPLC grade acetonitrile, methanol, and chloroform were purchased from Merck (Darmstadt, Germany). Ultrapure water was prepared by a Milli-Q Ultrapure water system (Millipore, Billerica, MA, USA). Seventy-eight metabolites used for evaluating annotation accuracy were supplied by Sigma-Aldrich (St. Louis, MO, USA) (Table S1). Nine stable isotope labeled internal standards (ISs) were used to normalize MS features in DI-nESI-HRMS. (Table S2). Carnitine C2:0-d_3_, carnitine C12:0-d_3_, and carnitine C16:0-d_3_ were purchased from International Laboratory (South San Francisco, CA, USA). Choline-d_4_, phenylalanine-d_5_ (Phe-d5), tryptophan-(indole-d_5_) (Trp-d_5_), leucine-d_3_ (Leu-d_3_), glutamine-d_5_, cholic acid-d_4_ (CA-d_4_), chenodeoxycholic acid-d_4_ (CDCA-d_4_) were purchased from Sigma-Aldrich (St. Louis, MO, USA).

### 2.2. Sample Information and Preparation

The serum samples were collected from 38 healthy control individuals and 31 type 2 diabetes (T2D) patients at The Second Hospital of Dalian Medical University. All enrolled participants had written consents and the research protocol (No. 2019(124)) was approved by the Ethics and Human Subjects Committee of the Second Hospital of Dalian Medical University. Detailed clinical data of the samples are summarized in Table S3.

A pooled sample was prepared by combining 10 μL aliquots from each serum sample as a quality control (QC) sample. The working solution of IS was mixed in methanol for subsequent protein precipitation of serum samples. Method linearity was assessed with the mixture of 10 metabolite standards at 6 different concentrate levels (Table S4).

For DI-nESI HRMS analysis, routine protein precipitation was first performed. Then, 200 μL of ice-cold methanol containing IS was added into a 1.5 mL Eppendorf tube containing 50 μL of a serum sample, followed by thorough vortexing for 1 min, and centrifuged for 10 min at 4 °C and 15,000× *g*. 220 μL of the supernatant was vacuum concentrated at 4 °C by CentriVap Centrifugal Vacuum Concentrators (Labconco, Kansas City, MO, USA). Then, the freeze-dried residue was diluted 20 times with methanol/water 2:1 (*v/v*) containing 0.1% formic acid. After vortexing for 1 min, the sample solution was centrifuged at 4 °C and 15,000× *g* for 10 min. Finally, the supernatant was transferred into 96-well plates for DI-nESI HRMS analysis.

### 2.3. DI-nESI HRMS Analysis

In DI-nESI HRMS analysis, TriVersa Nanomate chip electrospray system (Advion BioSciences, Ithaca, NY, USA) was coupled to a Q Exactive HF (Thermo Fisher Scientific, Rockford, IL, USA). For the NanoMate system, the voltage and gas pressure were set as 1.7 kV and 0.6 psi, respectively. For mass spectrometry acquisition, the capillary temperature was set at 270 °C and S-lens level was 50. The data of the full scan based on the spectral-stitching acquisition were acquired in positive ionization mode and the mass resolution at *m*/*z* 200 was 240,000, the scan windows were set as follows: *m*/*z* 65–235, *m*/*z* 225–315, *m*/*z* 305–355, *m*/*z* 345–395, *m*/*z* 385–435, *m*/*z* 425–475, *m*/*z* 465–515, *m*/*z* 505–562, *m*/*z* 552–609. Maximum IT was 200 ms and Microscan was set as 3. The total analysis time was 0.6 min per sample. The spectrum data type above all acquisition methods was set as centroid mode.

### 2.4. Data Processing

For DI-nESI-Orbitrap MS analysis, Xcalibur software (Thermo Fisher Scientific, Rockford, IL, USA) was used to visualize and process rawdata files. Theoretical formulas for MS features were generated according to the rules of elemental type (C, H, O, N, P, S), hydrogen/carbon ratios (0.4~5.1) and isotopic fine structure, and further filtration restricting elemental composition and reaction network analysis was performed using in-house Python scripts. The subsequent steps were processed including peak lists export, noise filtration (S/N > 10), peaks alignment (5 ppm), and blank reduction.

The MS feature intensities were normalized by ISs and only those features with a relative standard deviation (RSD) less than 30% in QC were retained for further statistical analysis. Finally, the peak table was used for statistical analyses, and the partial least squares-discriminant analysis (PLS-DA) was performed. Significantly differential metabolites between health and T2D diabetes were screened out by nonparametric tests (*p* < 0.05) and VIP > 1.

An initial reaction network was constructed using unique molecular formulas (seed nodes) extracted from the Human Metabolome Database (HMDB, https://hmdb.ca/metabolites) (accessed on 16 December 2022). Three filtering criteria were employed for the selection of seed metabolites. Criterion 1 included metabolites filtered by the biospecimen of “Blood” and the origin of “Endogenous”, criterion 2 included metabolites filtered by the origin of “Endogenous”, and criterion 3 included all metabolites in the HMDB without any filtering. After restricting the elemental composition to C, H, O, N, P, and S, non-redundant formulas within a mass range of 50~800 were retained. To establish edges between nodes, the differences between any two formulas were obtained. If they matched the pre-defined reactions listed in Table S5, edges between nodes were established. Next, all possible formula candidates of experimental features were generated based on their accurate mass. The formula differences between the candidates and the nodes in the initial reaction network were calculated using custom Python scripts. If the formula differences matched the pre-defined reactions, edges were connected between the possible formula candidates and the nodes in the initial reaction network. The topological parameters were obtained by network analysis. The molecular network was visualized using Cytoscape (version 3.8.0).

## 3. Results

### 3.1. Workflow of the Developed Method

**Figure 1:**
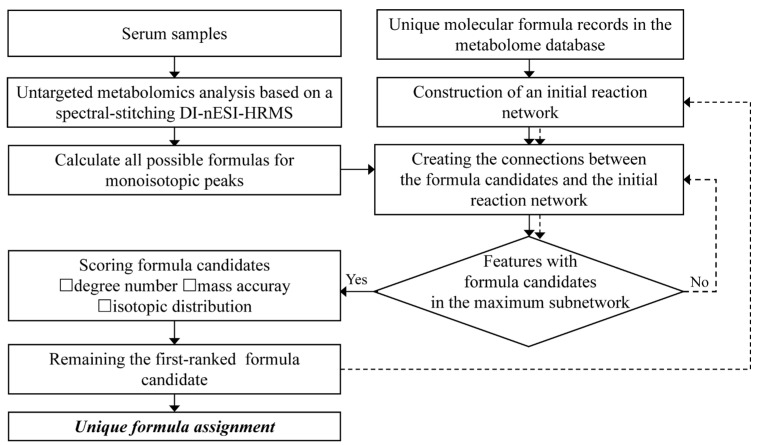
The workflow of a comprehensive serum metabolome analysis based on DI-nESI HRMS.

The entire workflow of the developed comprehensive characterization of serum metabolome is displayed in Figure 1. First, untargeted serum metabolome analysis was performed using spectral-stitching acquisition based on DI-nESI HRMS. Possible formula candidates for all the detected monoisotopic MS features were generated according to the rules outlined in the Data processing section with a mass accuracy of 2 ppm. A reaction network approach was then used to screen reliable formula candidates, using 76 predefined biological reactions as edge species (Table S5). The initial reaction network was constructed using unique molecular formula records in the metabolome database as seed nodes. The nodes were connected when their formula difference met with the predefined reactions. Then, the formula difference between all possible candidates and nodes in the initial reaction network was calculated. If the formula difference satisfies the predefined reaction, a connection is established between the candidate formula and seed nodes. For the monoisotopic MS features with multiple formula candidates connected with the initial reaction network, a scoring system was applied to select the top-ranked candidate as the unique formula. These unique formula candidates were newly-seeded nodes to integrate with the initial seeds and reconstructed the reaction network. For the monoisotopic MS features without any formula candidates connected to the initial reaction network, the formula candidates were screened using the reconstructed network again. This filtering process was repeated until no additional features were assigned with unique formulas. Finally, all the unique formula assignments were summarized.

### 3.2. The Method Establishment

To evaluate the mass accuracy of spectral-stitching DI-nESI HRMS, 78 reference metabolites dissolved in pure solvent (standard mixture) were analyzed (Table S1). A total of 101 monoisotopic MS features related to the standard mixture, including three adduct types ([M+H]^+^, [M+Na]^+^, and [M+K]^+^) were detected. As shown in Figure S1, the mass error distribution of the metabolites was mainly clustered within 2 ppm and near zero. Therefore, a mass error of 2 ppm was used in the following formula assignment.

**Figure 2:**
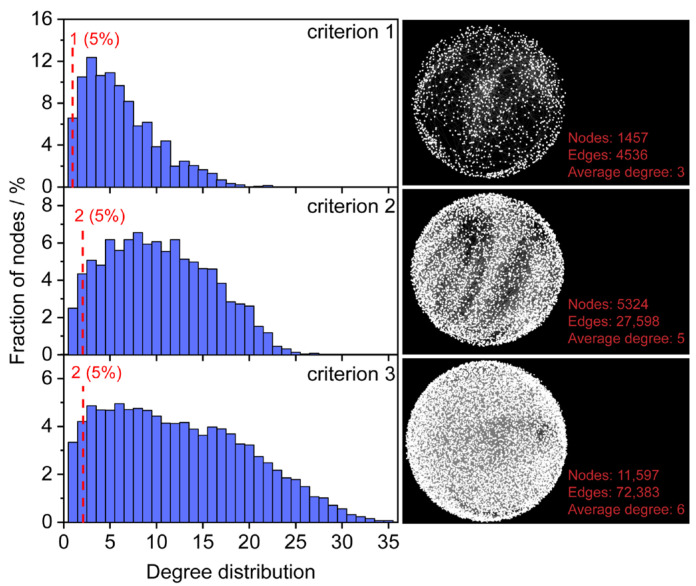
Degree distributions of seed nodes in the initial network. Criterion 1 represents endogenous metabolites from blood (1650), criterion 2 represents endogenous (5620) and criterion 3 represents all metabolites in the HMDB (12,351).

Metabolism in a biological system is highly interconnected through metabolic reactions. Thus, the relevance between potential formula candidates and known endogenous metabolites in a reaction network can be leveraged to estimate the reliability of the formula assignment. The HMDB is a comprehensive database that contains known human metabolites [11]. To improve the accuracy and coverage of formula assignments, metabolites were selected from the HMDB and served as seed nodes for constructing an initial reaction network. To investigate the impact of seed nodes on formula assignment, three filtering criteria were employed for obtaining “endogenous metabolites from blood” (criterion 1), “endogenous metabolites” (criterion 2), and “all metabolites” (criterion 3) in the HMDB database. A total of 1650, 5620, and 12,351 non-redundant formulas were obtained for criteria 1, 2, and 3, respectively. Three initial metabolic reaction networks were constructed, in which 1457/4536, 5324/27,598, and 11,597/72,383 nodes/edges were obtained using criteria 1, 2, and 3 (Figure 2).

Degree distributions of the three initial metabolic reaction networks were evaluated (Figure 2). With the increase of non-redundant formulas from 1650 to 12,351, the nodes display closer connections of the average degree from 3 to 6. The fractions of nodes with a degree value less than three were 17.1% (criterion 1), 6.8% (criterion 2), and 7.5% (criterion 3), respectively. The initial network constructed by endogenous metabolites (criterion 2) had the lowest proportion of low-degree nodes. Considering formula candidates with a high degree may present higher confidence, the cut-off values of the degree to the three initial reaction networks were set 2, 3, and 3, respectively.

The above standard mixture was used to assess three initial reaction networks. The overlap of the molecular formula between the standard mixture and metabolites in criteria 1, 2, and 3 was 58, 72, and 77, respectively (Figure S2). Using a mass accuracy of 2 ppm, 457 possible formula candidates for 101 features in the standard mixture were generated. All the possible formula candidates were further connected with three initial reaction networks. Among these possible formula candidates, 140 possible formula candidates corresponding to 98 features (criterion 1), 166 formula candidates corresponding to 101 features (criterion 2), and 198 possible formula candidates corresponding to 101 features have a network connection. A total of 119/140, 127/166, and 171/198 possible formula candidates had one more (criterion 1) and two more neighbor nodes (criteria 2 and 3). Through the network filter, the number of MS features with multiple formula candidates increased from 19 (criterion 1) to 23 (criterion 2) and 32 (criterion 3), respectively.

After filtering by the reaction network, there were still many monoisotopic MS features with multiple formula candidates. A scoring rule was established to further rank formula candidates using three alternative scoring criteria, including mass accuracy, isotope pattern, and degree of formula candidates (Equation (1)).

(1) $$\mathrm{Score}=W_{\mathrm{degree}}{\mathrm{Score}}_{\mathrm{degree}}-W_{m/z}{\mathrm{Score}}_{m/z}+W_{\mathrm{iso}}\;{\mathrm{Score}}_{\mathrm{iso}}$$

The *W*_degree_, *W_m/z_* and *W*_iso_ represent the weight coefficients of each criterion. Score*_m/z_* represents the mass accuracy score between experimental *m*/*z* (*m*/*z*_E_) and theoretical *m*/*z* (*m*/*z*_T_) values (Equation (2)):

(2) $${\mathrm{Score}}_{m/z}=\frac{\left|\left(m/z_{E}-m/z_{T}\right)\right|}{m/z_{E}\times 2}\times 10^{6}$$

Score_iso_ represents the similarity of isotopic distribution between experimental and theory isotopic patterns (Equation (3)):

(3) $${\mathrm{Score}}_{\mathrm{iso}}=0.5\times {\mathrm{Score}}_{mz}+0.5\times {\mathrm{Score}}_{\mathrm{int}}$$

In which ${\mathrm{Score}}_{mz}=1-\frac{|(m/z_{E}-m/z_{T})|/(m/z_{T})\times 10^{6}}{{\mathrm{Tolerance}}_{m/z}}$ and ${\mathrm{Score}}_{\mathrm{int}}=1-\frac{|({\mathrm{int}}_{E}-{\mathrm{int}}_{T})|/({\mathrm{int}}_{T})\times 10^{6}}{{\mathrm{Tolerance}}_{\mathrm{int}}}$. Where int_E_ and int_T_ are the experimental and theoretical relative intensity, respectively. Tolerance*_m/z_* and Tolerance_int_ represent the mass tolerance of 2 ppm and the relative intensity tolerance of 500%, respectively [38].

The effects of the weight coefficients on formula assignments were evaluated using the above standard mixture (Figure S3). If only accurate mass filtering (*W*_degree_ = 0, *W_m/z_* = 1, *W*_iso_ = 0) was used, the correct assignment rate for standards was 73.3~81.2%. If degree filtering was considered alone (with *W*_degree_ = 1, *W_m/z_* = 0, *W*_iso_ = 0), the correct assignment rate for standards increased to 85.1~87.1%. The combination of all three scoring criteria resulted in a correct assignment rate of approximately 90%. The optimal weight coefficients were determined to be *W*_degree_ = 0.5, *W_m/z_* = 0.3, and *W*_iso_ = 0.2.

**Figure 3:**
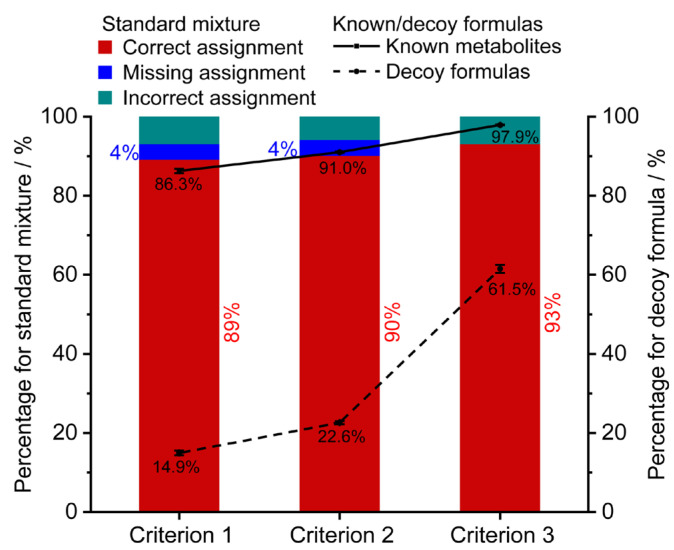
The effects of the initial networks on the assignment performance. The formula assignment results of the standard mixture (stacked column), 1000 blood metabolites (solid line), and 1000 decoy formulas (dash line). Criterion 1, 2, and 3 represent the initial network constructed by 1650 endogenous formulas from blood, 5620 endogenous formulas, and all the 12,351 formulas in the HMDB database, respectively. Error bars represent ±SE (*n* = 3).

The assignment performance of three initial reaction networks for the standard mixture was compared using the optimal weight coefficients (Figure 3). With the increase of non-redundant formulas from 1650 to 12,351, the correct assignment rate slightly increased from 89.1% to 93.1%. Using criteria 1 and 2, 4% of monoisotopic MS features were not retained any candidates because they lacked connections with the initial network. Furthermore, to assess the performance of the three initial networks, a target-decoy strategy was implemented. A set of 1000 known formulas presented in the HMDB database (blood matrix) and 1000 decoy formulas absent in the HMDB database (token from the ChEMBL database) were used. The line charts in Figure 3 demonstrate a similar filtering trend between the known/decoy formulas and the standard mixture. The percentage of formula assignment rates for known formulas increased slightly from 86.3%, 91.0% to 97.9% (solid line), while the percentage of formula assignment rates for decoy formulas significantly increased from 14.9%, 22.6% to 61.5% (dash line). The results implied that initial networks under criteria 1 and 2 exhibited more specific assignments for the biosample compared to criterion 3. As a result, the initial network consisting of 5620 endogenous metabolites (criterion 2) demonstrated superior assignment performance and was used for the subsequent characterization.

### 3.3. Method Validation

**Figure 4:**
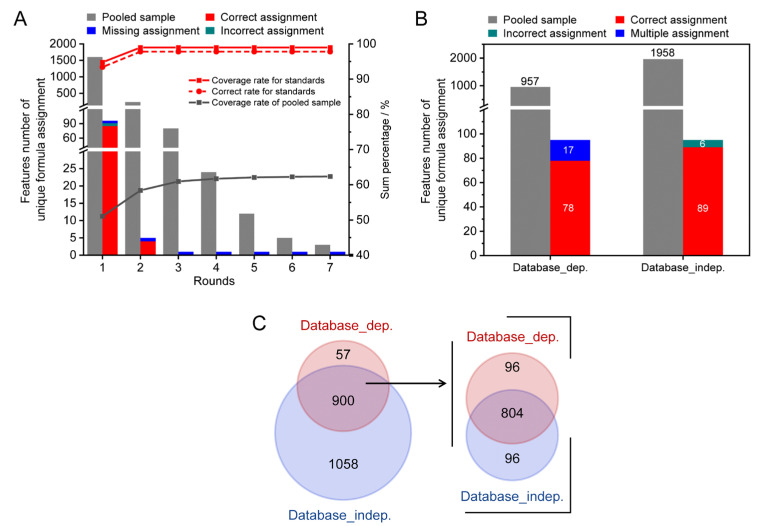
The method validation results using a spiked serum sample. (**A**) The coverage and accuracy rates of the developed method. (**B**) Comparison of assignment performance between the developed method and the database search. (**C**) The Venn diagram of assignment results of both methods for the spiked pooled serum sample.

A pooled serum sample spiked 78 reference standards was used for method validation. As shown in Figure 4A, 96 monoisotopic MS features were detected to be associated with 68 reference standards, which were assigned 353 potential formula candidates. Using the workflow described above, 95 out of the 96 monoisotopic MS features were successfully assigned with unique formula candidates through the two rounds of assignments (Figure 4A, stacked column). A total of 89 out of 95 assigned features were correctly allocated unique formulas. As a result, the coverage and accuracy rates were 98.9% (red solid line) and 93.6% (red dash line), respectively. For the spiked pooled serum sample, 3140 monoisotopic MS features were assigned with 19,474 possible formula candidates. Among these features, 1958 features were assigned with unique formula candidates after seven rounds of assignments (grey columns). The coverage rate was up to 62.3% (grey solid line). It is noteworthy that 18.1% of features were assigned between the second and seventh rounds.

The assignment performance of the developed method was compared with the conventional formula assignments method of searching *m*/*z* against the metabolome database (database-dependent method). The results are shown in Figure 4B. For the 68 spiked reference standards, the coverage rates of the two methods were similar (99%). However, the accuracy rates of unambiguous formula candidates were improved from 81.3% (78/96) to 92.7% (89/96) by the developed method. A total of 17.7 % (17/96) of MS features obtained multiple formula candidates by searching *m*/*z* against the HMDB (stacked column). For the spiked pooled serum sample, only 957 MS features were able to obtain unambiguous formula candidates by HMDB database search using a mass tolerance of 2 ppm, which yielded less than half the number of formulas compared to the developed method. The Venn diagram of the assigned monoisotopic MS features by two methods is shown in Figure 4C. It can be observed that the assigned monoisotopic MS features by the database search had good agreement with the developed method (900 out of 957). Among these 900 shared assigned monoisotopic MS features, about 89% (804/900) of MS features annotated consistent molecular formulas. The results indicated that the developed method showed high coverage and accuracy for formula assignment in the characterization of complex biosamples.

### 3.4. Application for Serum Metabolomic Analysis in Diabetes

To verify the practicability of the developed method, it was used to investigate the metabolic alterations in diabetes. Table S3 lists the detailed clinical information of participants corresponding to two groups of healthy control and diabetes patients. No significant differences except for blood glucose concentration were observed between the two groups. The repeatability of the developed acquisition method was measured using QC samples. The RSD values were estimated (Figure S4A). It shows that 86% of the detected MS features had RSD less than 30%, accounting for 96% of the sum peak area. Then, only those assigned features with an RSD of less than 30% in QC were subjected to further statistical analysis. The method linearities were evaluated using 10 reference metabolites (Table S4). The method exhibited good linearities with linear correlation coefficients (*R*^2^) of 0.9975–1, and linear ranges spanning two to four orders of magnitude. The results demonstrated that the current acquisition method was appropriate for serum metabolomics analysis.

Figure S4B represents a PLS-DA score plot of two groups. The separated clusters were observed between healthy control and patients. To further validate the classification models, the permutation test was performed (M = 200). The intercept values of *R*^2^ and *Q*^2^ were below 0.4 and −0.05, respectively, which indicated no overfitting of the PLS-DA model (Figure S4C).

**Figure 5:**
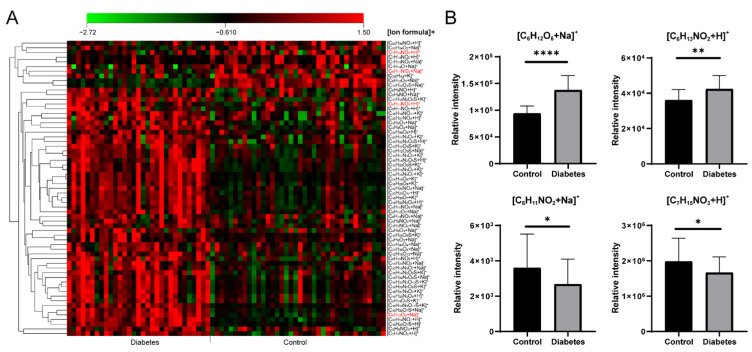
(**A**) The heat map of significantly changed ion formulas between control and diabetes groups, (**B**) Changes of [C_6_H_12_O_6_+Na]^+^, [C_6_H_13_NO_2_+H]^+^, [C_6_H_11_NO_2_+Na]^+^ and [C_7_H_15_NO_3_+H]^+^ between control and diabetes groups, respectively. **** *p* < 0.0001, ** *p* < 0.01, * *p* < 0.05.

A total of 57 significantly differential formulas were obtained between healthy control and T2D patients with VIP > 1 and *p* < 0.05 (Table S6). A heat map of the significantly changed metabolic features revealed good clustering between the control and T2D groups (Figure 5A). Four typical metabolites ([C_6_H_12_O_6_+Na]^+^, [C_6_H_13_NO_2_+H]^+^, [C_6_H_11_NO_2_+Na]^+^ and [C_7_H_15_NO_3_+H]^+^) are displayed in Figure 5B. Compared with the controls, significantly increased [C_6_H_12_O_6_+Na]^+^ (glucose), [C_6_H_13_NO_2_+H]^+^ (Leucine/Isoleucine) and decreased [C_6_H_11_NO_2_+Na]^+^ (pipecolinic acid), [C_7_H_15_NO_3_+H]^+^ (carnitine) in diabetes were observed. As shown in the clinical information (Table S3), blood glucose existed a significant difference between healthy and T2D groups, it was consistent with our result as [C_6_H_12_O_6_+Na]^+^. In the previous study, the abnormal concentrations of carnitines also appeared in the T2D groups owing to the fatty acid oxidation dysregulation [39,40], our result showed a decrease of [C_7_H_15_NO_3_+H]^+^ in T2D group. A positive association of branch-chain amino acids in diabetes had been concluded by modulating insulin secretion and leading to the pancreaticb-cell exhaustion [41,42]. Leucine/isoleucine (C_6_H_13_NO_2_) recognized as branch chain amino acids had a robust association with the risk of T2D, a similar trend with [C_6_H_13_NO_2_+H]^+^ is shown in Figure 5B. In addition, Ouyang et al. observed that pipecolinic acid (C_6_H_11_NO_2_) was decreased in T2D patients in a Chinese prospective cohort study [43].

## 4. Discussion

DI-nESI HRMS is a promising high-throughput analytical method for large-scale cohort metabolomic study [44,45]. High coverage and reliable assignment of MS features is a critical prerequisite for comprehensive metabolomics analysis [46]. In the present study, we aimed to develop a comprehensive metabolomics analysis method in direct-infusion high-resolution mass spectrometry. Our result revealed that based on a reaction network combing with mass accuracy and isotopic distribution, more MS peaks could be assigned with a confident unique formula.

For untargeted metabolomics analysis based on DI-HRMS, the most widely used approach for formula assignments was searching *m*/*z* against metabolome databases. According to the performance of mass resolution (Rs) and mass accuracy in the high-resolution mass spectrometer, appropriate mass tolerance parameter was obtained, such as 10~20 ppm for time-of-flight (Rs on the order of 35,000), 5~15 ppm for orbitrap (Rs on the order of 200,000 at *m*/*z* 200) and 0.5 ppm for Fourier transform ion cyclotron mass spectrometers (Rs 900,000 at *m*/*z* 200) [47,48]. However, even with tremendous advances in database size and scape, the identification of metabolites still is a primary challenge in the field [49,50].

The relationship between metabolites attracted wide attention for formula identification in untargeted DI ultrahigh resolution MS. Reaction network approach based on mass difference had been used for MS feature assignments, in which experimental features as nodes and chemical reactions as edges [19,22]. However, reliable and enough experimental features need to be first assigned as initial seed nodes. It is difficult to achieve for the untargeted metabolomics analysis using HRMS. Instead of only experimental features used for the reaction network, the formula records from the metabolome database were used to construct the initial reaction network in this study. The connections between formula candidates and the initial reaction network were established for filtering multiple candidates. The assignment performance was improved due to sufficient and accurate known formulas from the initial network. Furthermore, using assigned unambiguous formula candidates as newly added seeds to reconstruct networks, multiple rounds of annotation were designed to further improve annotation efficiency (1.22-fold increase after 7 rounds in this study). Integration of mass accuracy and isotopic pattern filter resulted in a further improvement in assignment accuracy.

Although the developed method demonstrated more than twice the number of unique assigned formulas for a pooled serum sample compared to the database search, still about 38% of MS features could not obtain unambiguous formula candidates. The false negatives are possibly caused by the initial network filter. To avoid false positives, the initial network in this study was constructed using only the most common metabolic reactions and endogenous formulas from the HMDB database. Furthermore, the excellent assignment performance for serum untargeted metabolic features is partly attributed to the comprehensive HMDB database for the construction of the initial reaction network. Nevertheless, for the less studied objects, the method performance may be suboptimal because the initial network can’t be established or the initial network size is too small. Furthermore, it is essential to accurately confirm the structures of differential metabolites and then determine key differential metabolites from them in subsequent studies.

## 5. Conclusions

A comprehensive serum metabolome characterization method in DI-nESI HRMS was proposed in this study. High coverage, high confidence, and database-independent formula assignments were achieved by reaction network combined with mass accuracy and isotopic pattern filter. A total of 1958 monoisotopic features were assigned the unique formula in a pooled serum sample, while only 957 were annotated by searching *m*/*z* against HMDB. This method has the advantages of comprehensive formula assignment and high-speed acquisition, it has great application potential in large-scale cohort metabolomic studies.

## Data Availability

Data will be made available upon request from the corresponding author. The data are not publicly available due to privacy.

## Supplementary Materials

The following supporting information can be downloaded at: https://www.mdpi.com/article/10.3390/metabo13030460/s1, Figure S1: Mass error distribution of metabolites standards; Figure S2: The overlap between the standards formulas and seed nodes in three initial reaction networks; Figure S3: Weight coefficients optimization for the degree, mass error and isotopic distribution; Figure S4: (A) Estimation of detection repeatability. (B) PLS-DA scores plot of healthy and diabetes groups. (C) Cross-validation plots of the PLS-DA model; Table S1: Information of metabolites standards; Table S2: Information of internal standards; Table S3: Clinical information of participants; Table S4: Information of 10 standards for linearity; Table S5. Reactions types; Table S6. Significantly changed features between healthy and T2D groups.
