# Supplementary material for: A Strategy for Uncovering the Serum Metabolome by Direct-Infusion High-Resolution Mass Spectrometry
Source: Metabolites. 2023 Mar 22;13(3):460. doi: 10.3390/metabo13030460 (PMC10057860; doi:10.3390/metabo13030460)
Supplement: Supplementary file 1 [file metabolites-13-00460-s001.zip › metabolites-2292157-supplementary.pdf]

Supporting Information

A Strategy for Uncovering the Serum Metabolome by Direct-Infusion High-resolution Mass Spectrometry

Xiaoshan Sun [1,2,3], Zhen Jia [1,3,4], Yuqing Zhang [1,3,5], Xinjie Zhao [1,2,3], Chunxia Zhao [1,2,3], Xin Lu [1,2,3,*] and Guowang Xu [1,2,3,*]

[1] CAS Key Laboratory of Separation Science for Analytical Chemistry, Dalian Institute of Chemical Physics, Chinese Academy of Sciences, Dalian 116023, China; sunxiaoshan2015@dicp.ac.cn (X.S.); jiazhen@dicp.ac.cn (Z.J.); yuqingzhang@dicp.ac.cn (Y.Z.); xj_zhao@dicp.ac.cn (X.Z.); zhaocx@dicp.ac.cn (C.Z.)
[2] University of Chinese Academy of Sciences, Beijing 100049, China
[3] Liaoning Province Key Laboratory of Metabolomics, Dalian 116023, China
[4] Department of Cell Biology, College of Life Sciences, China Medical University, Shenyang 110122, China
[5] Zhang Dayu School of Chemistry, Dalian University of Technology, Dalian 116024, China
* Correspondence: luxin001@dicp.ac.cn (X.L.); xugw@dicp.ac.cn (G.X.)

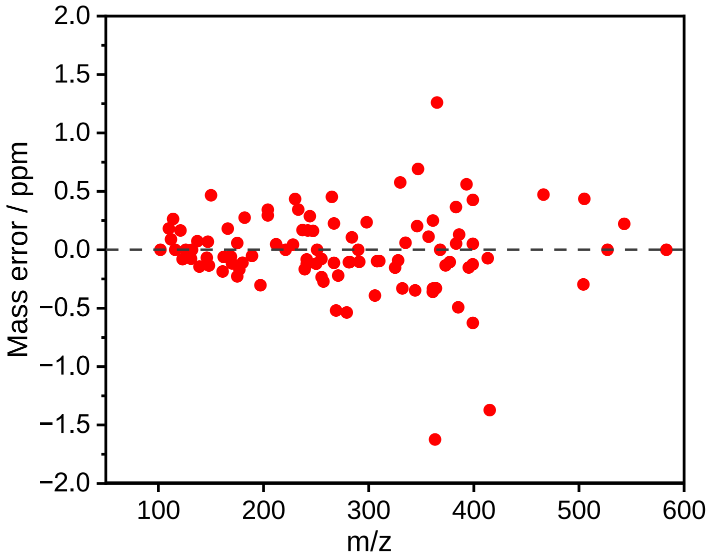

Figure S1. Mass error distribution of the 101 monoisotopic MS features related to the reference compounds in the standard mixture

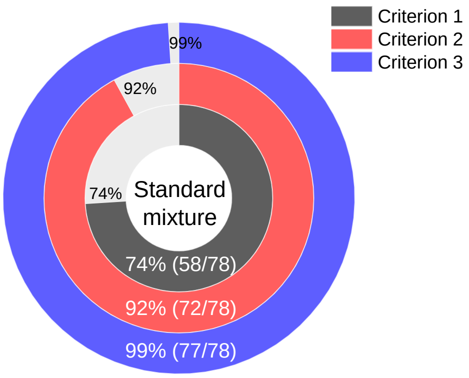

Figure S2. The overlap between standards and metabolites filtering using criterion 1, criterion 2 and criterion 3, respectively. Criteria 1, 2 and 3 represent endogenous metabolites from blood, endogenous metabolites, and all metabolites in the HMDB database, respectively.

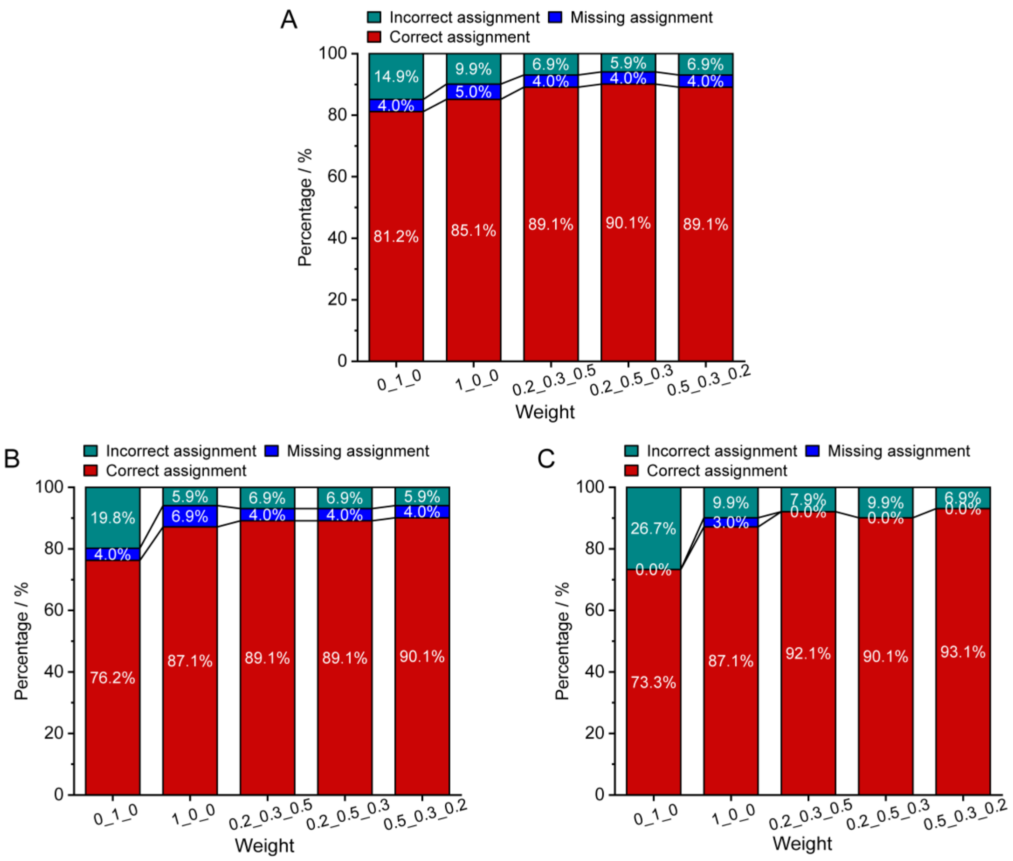

Figure S3. The effects of the weight coefficients on formula assignments in the standard mixture using three initial networks constructed by criterion 1 (A), criterion 2 (B), and criterion 3 (C). $W_{\text{degree}}\_W_{m/z}\_W_{\text{iso}}$ of the *x*-axis represents weight coefficients of degree, mass accuracy, and isotopic distribution, respectively.

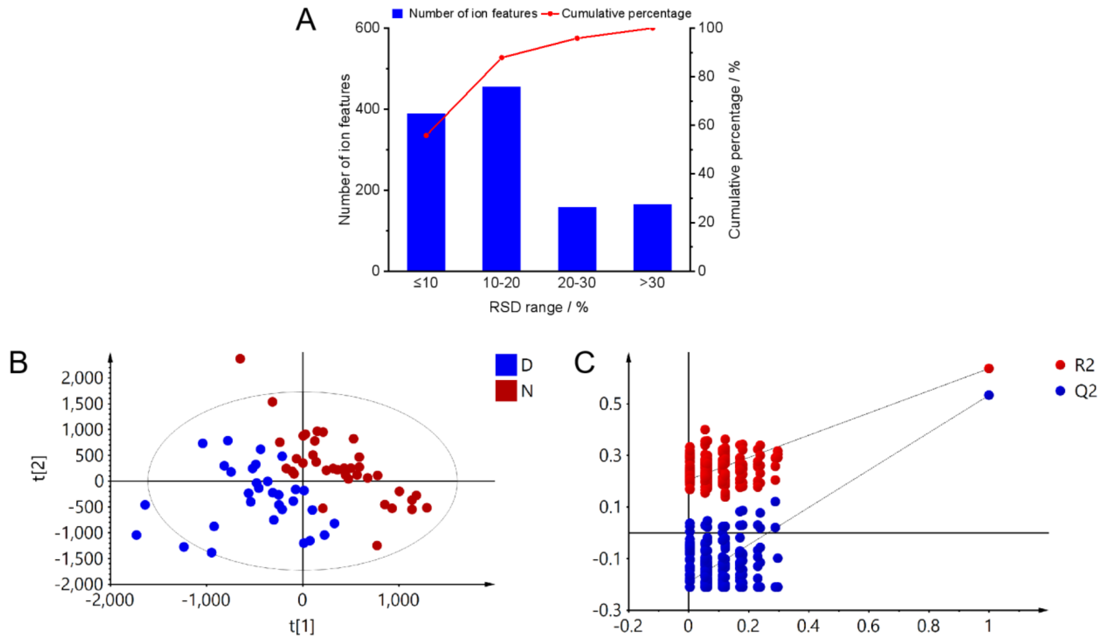

Figure S4. (A) The RSD distribution of MS features in QCs. (B) PLS-DA score plot of healthy control (red dots) and diabetes patients (blue dots). (C) Cross-validation plots of the PLS-DA model with 200 times permutation tests.

Table S1. Detailed information on 78 metabolites standards

No	Name	Formula	Monoisotopic mass
1	1-Aminocyclopropane-1-Carboxylate	C4H7NO2	101.0477
2	2-Aminophenol	C6H7NO	109.0528
3	Cytosine	C4H5N3O	111.0433
4	Creatinine	C4H7N3O	113.0589
5	L-Proline	C5H9NO2	115.0633
6	Purine	C5H4N4	120.0436
7	Nicotinamide	C6H6N2O	122.0480
8	Creatine	C4H9N3O2	131.0695
9	Hypoxanthine	C5H4N4O	136.0385
10	Urocanate	C6H6N2O2	138.0429
11	4-Guanidinobutanoate	C5H11N3O2	145.0851
12	Dimethylbenzimidazole	C9H10N2	146.0844
13	3-Methyl-2-Oxindole	C9H9NO	147.0684
14	3-Methyladenine	C6H7N5	149.0701
15	Tryptamine	C10H12N2	160.1000
16	5-Methylcytosine Hydrocloride	C5H7N3O	125.0589
17	L-Phenylalanine	C9H11NO2	165.0790
18	N-Acetylputrescine	C6H14N2O	130.1106
19	Amylose	C14H26O11	370.1475
20	N(Pai)-Methyl-L-Histidine	C7H11N3O2	169.0851
21	N-Omega-Methyltryptamine	C11H14N2	174.1157
22	Indole-3-Acetamide	C10H10N2O	174.0793
23	L-Tyrosine	C9H11NO3	181.0739
24	Deoxycarnitine	C7H15NO2	145.1103
25	Nalpha-Acetyl-L-Lysine	C8H16N2O3	188.1161
26	L-Carnitine	C7H15NO3	161.1052
27	3-Methoxytyramine	C9H13NO2	167.0946
28	Ethyl 3-Indoleacetate	C12H13NO2	203.0946
29	N-Acetyl-L-Phenylalanine	C11H13NO3	207.0895
30	Serotonin	C10H12N2O	176.0950
31	Dethiobiotin	C10H18N2O3	214.1317
32	Omega-Hydroxydodecanoic Acid	C12H24O3	216.1725
33	N-Acetylserotonin	C12H14N2O2	218.1055
34	5-Hydroxy-L-Tryptophan	C11H12N2O3	220.0848
35	Nepsilon,Nepsilon,Nepsilon-Trimethyllysine	C9H20N2O2	188.1525
36	Deoxycytidine	C9H13N3O4	227.0906
37	Deoxyuridine	C9H12N2O5	228.0746
38	Melatonin	C13H16N2O2	232.1212
39	L-Tryptophanamide	C11H13N3O	203.1059
40	O-Acetyl-L-Carnitine	C9H17NO4	203.1158
41	Pyridoxamine	C8H12N2O2	168.0899
42	Thymidine	C10H14N2O5	242.0903
43	Cytidine	C9H13N3O5	243.0855
44	Uridine	C9H12N2O6	244.0695
45	Biotin	C10H16N2O3S	244.0882
46	N-Acetyl-D-Tryptophan	C13H14N2O3	246.1004
47	3-Methoxy-L-Tyrosine	C10H13NO4	211.0845

No	Name	Formula	Monoisotopic mass
48	Palmitoleic Acid	C16H30O2	254.2246
49	1-Methyl-6,7-Dihydroxy-1,2,3,4-Tetrahydroisoquinoline	C10H13NO2	179.0946
50	Inosine	C10H12N4O5	268.0808
51	Gamma-Linolenic Acid	C18H30O2	278.2246
52	Linoleate	C18H32O2	280.2402
53	1-Methyladenosine	C11H15N5O4	281.1124
54	Guanosine	C10H13N5O5	283.0917
55	Ophthalmic Acid	C11H19N3O6	289.1274
56	5'-Methylthioadenosine	C11H15N5O3S	297.0896
57	Rac-Glycerol 1-Myristate	C17H34O4	302.2457
58	L-Anserine	C10H16N4O3	240.1222
59	2'-Deoxycytidine 5'-Monophosphate	C9H14N3O7P	307.0569
60	N-Acetylneuraminate	C11H19NO9	309.1060
61	Uridine-5-Monophosphate	C9H13N2O9P	324.0359
62	Cytidine 2',3'-Cyclic Mono-Phosphate	C9H12N3O7P	305.0413
63	Nicotinamide Mononucleotide	C11H15N2O8P	334.0566
64	5-Aminoimidazole-4-Carboxamide-1-Beta-D-Ribofuranosyl 5'-Monophosphate	C9H15N4O8P	338.0628
65	Lauroylcarnitine	C19H37NO4	343.2723
66	Rosmarinic Acid	C18H16O8	360.0845
67	Cortisone	C21H28O5	360.1937
68	Cortisol	C21H30O5	362.2093
69	Guanosine 3',5'-CyclicMonophosphate	C10H12N5O7P	345.0474
70	Deoxycorticosterone Acetate	C23H32O4	372.2301
71	Riboflavin	C17H20N4O6	376.1383
72	Bis(2-Ethylhexyl)Phthalate	C24H38O4	390.2770
73	Xanthosine 5'-Monophosphate	C10H13N4O9P	364.0420
74	Guanosine 5'-Monophosphate	C10H14N5O8P	363.0580
75	Glycocholate	C26H43NO6	465.3090
76	S-(5'-Adenosyl)-L-Methionine	C15H22N6O5S	398.1372
77	D-(+)-Raffinose	C18H32O16	504.1690
78	Biliverdin	C33H34N4O6	582.2478

Table S2. Detailed information on internal standards

Reference Compounds	m/z (positive mode)	Formula	Concentration (μg/mL)
Phe-d_5	171.11764	$C_9H_6D_5NO_2$	2.2
Trp-d_5	210.12854	$C_{11}H_7D_5N_2O_2$	4.0
Leu-d_3	135.12074	$C_6H_{10}D_3NO_2$	2.4
Glutamine-d_5	152.10780	$C_5H_5D_5N_2O_3$	5.0
CDCA-d_4	379.31448	$C_{24}H_{36}D_4O_4$	10
Choline-d_4	108.13210	$C_5H_9D_4NO$	0.3
Carnitine C2:0-d_3	207.14186	$C_9H_{14}D_3NO_4$	0.3
Carnitine C12:0-d_3	347.29836	$C_{19}H_{34}D_3NO_4$	0.3
Carnitine C16:0-d_3	403.36097	$C_{23}H_{42}D_3NO_4$	0.3

Table S3. Clinical information of participants

The clinical parameters	Diabetes	Control
Gender (male/female)	23/8	27/11
Age (years)	58±9	55±9
BMI (kg/m2)	22.66±6.42	23.03±2.35
Systolic BP (mm Hg)	124±9	122±9
Diastolic BP (mm Hg)	76±6	75±7
Blood glucose (mmol/L)	9.01±2.10	5.58±0.42
TG (mmol/L)	4.79±0.84	4.96±0.67
TG (mmol/L)	1.75±0.71	1.39±0.41
HDL-c (mmol/L)	1.24±0.27	1.34±0.24
LDL-c (mmol/L)	2.61±0.74	2.78±0.5

The clinical parameters are presented as mean ± SD

Table S4. The results of method linearity

Compound ID	m/z	Linearity	Linear range ng/ml	R^2
Phenylalanine	166.08625	y=0.1717x+1.0012	5-25000	1
Tryptophan	205.09715	y=1.8661x-409.68	50-25000	0.9996
Leucine	132.10190	y=1.4129x+132.19	5-25000	0.9999
Cholic acid	409.29485	y=0.0002x-0.1034	250-25000	0.9975
Methionine-(methyl)	150.05833	y=0.1699x+14.402	5-25000	0.9999
Pro-leu	229.15467	y=0.1503x+0.8896	25-25000	0.9998
Phe-glutamine	265.11828	y=0.0654x+2.7412	5-25000	1
Methyl-glutamine	162.07608	y=0.0588x-3.884	50-25000	0.9999
LPC 16:0	496.33977	y=0.0407x-7.1806	50-5000	0.9979
Carnitine C8:0-d3	291.23576	y=8.1911x-90.902	5-25000	0.9999

Table S5. Seventy-six commonly encountered reactions in biological systems

Reaction Type	Description	
H2	dehydrogenation	hydrogenation
CH2	demethylation	methylation
NH	loss of NH	addition of NH
O	loss of oxygen	Oxidation
NH3	loss of ammonia	addition of ammonia
H2O	loss of water	addition of water
CO	loss of CO	addition of CO
C2H4	loss of C2H4	addition of C2H4
C2H2O	Deacetylation	Acetylation
CO2	loss of CO2	addition of CO2
C2H3NO	loss of glycine	glycine conjugation
SO3	loss of sulfate	sulfate conjugation
HPO3	loss of Phosphate	addition of Phosphate
C4H3N3	loss of Cytosine	addition of Cytosine
C4H2N2O	loss of Uracil	addition of Uracil
C3H5NOS	loss of cysteine	cysteine conjugation
C2H5NO2S	loss of taurine	taurine conjugation
C5H4N2O	loss of Thymine	addition of Thymine
C3H5NO2S	loss of S-cysteine	S-cysteine conjugation
C5H8O4	loss of D-ribose	addition of D-ribose
C5H3N5	loss of Guanine	addition of Guanine
C7H13NO2	loss of Carnitine	addition of Carnitine
C5H7NO3S	loss of N-acetyl-S-cysteine	addition of N-acetyl-S-cysteine
C6H10O5	loss of Hexose	addition of Hexose
C6H8O6	loss of glucuronic acid	addition of glucuronic acid
C10H12N2O4	loss of Thymidine	addition of Thymidine
C9H11N3O4	loss of Cytidine	addition of Cytidine
C9H10N2O5	loss of Uridine	addition of Uridine
C16H30O	loss of Palmitic acid	addition of Palmitic acid
C6H11O8P	loss of Glucose-6-phosphate	addition of Glucose-6-phosphate
C10H11N5O3	loss of Adenosine	addition of Adenosine
C10H11N5O4	loss of Guanosine	addition of Guanosine
C10H15N3O5S	loss of Glutathione	addition of Glutathione
C10H15N3O6S	loss of S-Glutathione	addition of S-Glutathione
C12H20O10	loss of di-hexose	addition of di-hexose
C18H30O15	loss of tri-hexose	addition of tri-hexose
SH→SO3H	Sulfonic acid to Thiol	Thiol to Sulfonic acid
(C5H5N5 - H2O)	loss of Adenine	addition of Adenine

Table S6. The significantly changed features between healthy control and T2D patients

Neutral formula	m/z	Adduct type	*P*-value	Fold change (T2D/control)	
C9H14O2S	225.03415	$[M+K]^+$	4.76E-03	1.49	↑
C8H15NO6	244.07896	$[M+Na]^+$	1.89E-08	1.59	↑
C6H15NO6	198.09689	$[M+H]^+$	1.83E-05	2.18	↑
C6H13NO2	132.10178	$[M+H]^+$	1.57E-03	1.17	↑
	154.0836	$[M+Na]^+$	4.93E-02	1.16	↑
C6H12O6	203.05231	$[M+Na]^+$	2.34E-11	1.46	↑
C6H10O5	185.04175	$[M+Na]^+$	2.59E-07	1.63	↑
C5H9NO4	148.06021	$[M+H]^+$	5.74E-03	1.22	↑
C5H9NO2	138.05238	$[M+Na]^+$	2.73E-02	1.23	↑
C5H9NO	100.07552	$[M+H]^+$	5.53E-03	1.40	↑
	122.05748	$[M+Na]^+$	4.09E-03	1.44	↑
C5H11NO2	118.08608	$[M+H]^+$	2.81E-02	1.10	↑
C4H8O4	143.03131	$[M+Na]^+$	3.22E-06	1.54	↑
C4H8O3	127.03638	$[M+Na]^+$	9.17E-03	1.86	↑
C4H6O3	125.02076	$[M+Na]^+$	2.81E-02	1.25	↑
C3H7NO2	90.05489	$[M+H]^+$	3.90E-05	1.23	↑
	112.03671	$[M+Na]^+$	4.09E-03	1.26	↑
C3H6O3	113.02072	$[M+Na]^+$	1.71E-03	1.27	↑
C34H56O6	561.4137	$[M+H]^+$	4.08E-04	1.71	↑
C24H18O6	441.07416	$[M+K]^+$	1.12E-09	2.71	↑
C21H40O4	379.28149	$[M+Na]^+$	3.79E-03	1.63	↑
C21H38O4	377.26587	$[M+Na]^+$	2.46E-03	1.57	↑
C20H21NO6	372.14307	$[M+H]^+$	7.50E-05	1.52	↑
C20H20O12	453.10309	$[M+H]^+$	2.76E-07	1.58	↑
C20H19NO7	386.12286	$[M+H]^+$	4.50E-11	2.17	↑
C20H17N3O8	466.06418	$[M+K]^+$	2.10E-05	1.61	↑
C19H22N2O10S	509.06198	$[M+K]^+$	5.91E-07	2.32	↑
C18H22O7S	383.11551	$[M+H]^+$	1.85E-12	2.11	↑
	405.09744	$[M+Na]^+$	1.42E-11	2.26	↑
C18H22N2O8	395.14456	$[M+H]^+$	3.78E-08	1.61	↑
C18H20N2O9	409.12372	$[M+H]^+$	4.27E-04	1.54	↑
C18H20N2O8S	463.05612	$[M+K]^+$	2.00E-06	2.40	↑
C17H16N2O5S	399.04034	$[M+K]^+$	6.22E-06	2.14	↑
C16H30O7	373.16263	$[M+K]^+$	1.11E-07	1.70	↑
C16H28O8	387.14175	$[M+K]^+$	9.71E-08	1.73	↑
C16H21N3O7	406.10095	$[M+K]^+$	2.54E-06	2.52	↑
C16H20N4O9S	467.08347	$[M+Na]^+$	7.12E-07	1.47	↑
C16H19NO11	440.05942	$[M+K]^+$	1.36E-04	1.65	↑
C16H19N3O11S	500.03632	$[M+K]^+$	1.50E-10	2.82	↑
C16H16N6O7	443.0712	$[M+K]^+$	8.89E-10	2.81	↑
C15H22N2O9S	407.11139	$[M+H]^+$	4.20E-06	1.76	↑
C15H14N2O8S	383.05371	$[M+H]^+$	5.55E-07	1.75	↑

Neutral formula	m/z	Adduct type	*P*-value	Fold change (T2D/control)	
C15H14N2O5S	373.02454	$[M+K]^+$	2.17E-05	1.65	↑
C13H22O5S	329.08142	$[M+K]^+$	9.02E-06	2.97	↑
C13H15N3O4	316.06943	$[M+K]^+$	8.10E-09	2.01	↑
C13H12O3S	271.03967	$[M+Na]^+$	3.80E-07	1.49	↑
	287.01373	$[M+K]^+$	9.02E-06	1.54	↑
C12H25NO8	334.14682	$[M+Na]^+$	4.05E-08	1.93	↑
C12H20O5S	315.06589	$[M+K]^+$	1.08E-08	1.93	↑
C12H18O13	393.06265	$[M+Na]^+$	2.29E-05	1.48	↑
C11H23NO8	320.13126	$[M+Na]^+$	1.67E-06	1.90	↑
C11H11N3O3	272.04312	$[M+K]^+$	1.65E-07	1.55	↑
C10H19N3O7	316.11124	$[M+Na]^+$	2.03E-04	1.63	↑
C7H15NO3	162.11226	$[M+H]^+$	3.18E-02	0.84	↓
C7H14O	137.09354	$[M+Na]^+$	3.13E-02	0.82	↓
C7H13NO2	144.10172	$[M+H]^+$	4.96E-04	0.30	↓
	166.08366	$[M+Na]^+$	6.92E-04	0.28	↓
C6H12O5	187.05737	$[M+Na]^+$	1.68E-11	0.26	↓
C6H11NO2	152.06796	$[M+Na]^+$	3.48E-02	0.74	↓
C28H44	419.3064	$[M+K]^+$	1.93E-02	0.94	↓
C21H34O2	341.24478	$[M+Na]^+$	2.73E-02	0.76	↓
C20H39NO4	358.29471	$[M+H]^+$	2.81E-02	0.70	↓
C13H12O2S	255.04477	$[M+Na]^+$	9.83E-12	0.22	↓
